# Evaluation of the etiology of epilepsy and/or developmental delay in children via next-generation sequencing: a single-center experience

**DOI:** 10.3389/fped.2025.1471965

**Published:** 2025-02-27

**Authors:** Handan Kava, Ozlem Akgun-Dogan, Ahmet Yesilyurt, Yasemin Alanay, Ugur Isik

**Affiliations:** ^1^ Department of Pediatrics, School of Medicine, Acibadem Mehmet Ali Aydinlar University, Istanbul, Türkiye; ^2^ Division of Pediatric Genetics, Department of Pediatrics, School of Medicine, Acibadem Mehmet Ali Aydinlar University, Istanbul, Türkiye; ^3^ Acibadem Mehmet Ali Aydinlar University Rare Diseases and Orphan Drugs Application and Research Center (ACURARE), Acibadem Mehmet Ali Aydinlar University, Istanbul, Türkiye; ^4^ Department of Transitional Medicine, Health Sciences Institute, Acibadem Mehmet Ali Aydinlar University, Istanbul, Türkiye; ^5^ Acibadem Labgen Genetic Diagnosis Center, Istanbul, Türkiye; ^6^ Department of Genome Studies, Health Sciences Institute, Acibadem Mehmet Ali Aydinlar University, Istanbul, Türkiye; ^7^ Division of Pediatric Neurology, Department of Pediatrics, School of Medicine, Acibadem Mehmet Ali Aydinlar University, Istanbul, Türkiye

**Keywords:** epilepsy, developmental delay, next-generation sequencing, pediatric neurology, pediatric geneticist

## Abstract

**Background:**

We aimed to understand the genetic etiology in children presenting with epilepsy and/or developmental delay by using next-generation sequencing (NGS).

**Materials and methods:**

We included children presenting to our pediatric neurology clinic with a diagnosis of epilepsy and/or developmental delay between January 2019 and December 2021. We evaluated the patients using the NGS equipment in our genetic laboratory.

**Results:**

In total, 90 patients were included in the study. Twenty (34.4%) out of 58 patients who had undergone whole-exome sequencing (WES) had pathogenic or likely pathogenic (P/LP) variants and 11 (18.9%) had variants of unknown significance (VUS). Five (41.6%) out of 12 patients who had undergone whole-genome sequencing had P/LP variants and 5 (41.6%) had VUS. Eleven (55%) out of 20 patients who had undergone WES and chromosomal microarray had P/LP variants and 2 (10%) had VUS. Twenty-six novel variants were described. Twelve patients (13.3%) were diagnosed using a known specific treatment.

**Conclusion:**

NGS aids in precisely diagnosing children with epilepsy and/or developmental delay. Furthermore, it provides a correct prognosis, specific treatment methods, and a multidisciplinary approach.

## Introduction

The incidence of epilepsy in children ranges from 0.5 to 8 per 1,000 individuals (1–4). Its estimated lifetime prevalence is 1%, and the highest incidence is observed during the first year of life (5). Various developmental disorders, such as developmental delay (DD), intellectual disability (ID), autism spectrum disorder (ASD), and attention deficit and hyperactivity disorder (ADHD) are more common in children with epilepsy (6).

In developed countries, the absence of expected psychomotor development is observed in approximately 1% of children (7). DD and ID may occur alone or in conjunction with congenital malformations or neurological findings such as epilepsy, hypotonia, ataxia, electroencephalogram (EEG) abnormalities, and behavioral problems such as ASD and ADHD. DD and ID are phenotypically and genetically heterogeneous, and a specific diagnosis cannot be reached in most cases. Therefore, genetic tests play a crucial role in determining the cause of abnormality in this patient group (8).

Next-generation sequencing (NGS) technologies enable the reliable identification of candidate genes responsible for epilepsy and DD in a single, cost-effective, and time-efficient process (9). There are compelling reasons to make a specific diagnosis in a child presenting with DD. Diagnosis permits families to understand the long-term prognosis, access special education services, and learn about potential treatment options. Moreover, it is crucial for counseling parents regarding recurrence risk, prenatal diagnosis, and preimplantation genetic diagnosis. Furthermore, a diagnosis can help parents access research studies and connect with other families facing similar challenges (10).

Chromosomal microarray (CMA) has long been utilized as a first-line genetic test in children with DD and ID. With the advancement of NGS techniques, whole-exome sequencing (WES) and whole-genome sequencing (WGS) have become pivotal diagnostic tools for children with DD and ID. The American College of Medical Genetics and Genomics (ACMG) guidelines strongly recommend considering WES/WGS as a first- or second-line test for patients with DD and ID (10).

NGS has unveiled new candidate genes implicated in the pathogenesis of epilepsy. Ion channels play a crucial role in maintaining proper neuronal excitability; these include voltage-gated sodium, potassium, calcium, and chloride ion channels, and ligand-gated acetylcholine receptor-mediated ion channels. Mutations in genes encoding components of these ion channels lead to ion channel dysfunction, known as channelopathy, and form the basis for the development of epilepsy syndromes. Genetic changes causing channelopathies may contribute to the pathogenesis of epilepsy via the gain or loss of ion channel function (11).

Early genetic testing and diagnosis are foundational for accurate treatment of epilepsy. However, they should be complemented with functional tests to explore the characteristics of the pathophysiological mechanisms and the functional impact underlying the detected variant. Specifically in ion channel disorders, understanding gain-of-function mutations and loss-of-function mutations is critical when making therapeutic decisions (12, 13).

Experience with most of the above mentioned treatments is limited to a relatively small number of patients. With a better understanding of the pathogenesis and cellular electrophysiology of genetic epilepsies, more treatment options are likely to emerge (14).

## Materials and methods

This retrospective cohort study included 90 patients aged 0–18 years, who were observed at the Acibadem Altunizade Hospital Pediatric Neurology Outpatient Clinic between January 2019 and December 2021 and had been diagnosed with epilepsy and/or DD. These patients had undetermined etiology despite undergoing biochemical and metabolic tests and magnetic resonance imaging (MRI). The genetic analyses were performed as part of the diagnostic process when patients first presented for evaluation. Informed consent for genetic testing was obtained by the accredited diagnostic laboratory prior to the tests. The WES and CMA results were processed at the Acibadem Labgen Genetic Diseases Evaluation Center, and the WGS results were obtained from the Centogene Laboratory (Germany).

Retrospective evaluation of the WES, CMA, and WGS results was conducted in collaboration with the Pediatric Genetics Department of Acibadem University. Patient medical records were retrospectively reviewed; these encompassed their medical history; background; family history; neurological symptoms; motor and mental developmental history; neurological examination findings; liver and kidney function test results; metabolic screening test, EEG, and MRI results; antiepileptic treatments; and genetic diagnoses.

Ethical approval for the retrospective study was obtained from the Scientific Research Ethics Committee of Acibadem University School of Medicine (Decision No: 2021/25/26).

### Whole-exome sequencing

Genomic DNA isolation was performed using peripheral blood leukocytes. Enrichment was performed using the Twist® Human Core Exome kits. Prepared libraries were sequenced using the Novaseq 6000 (Illumina) instrument. Using the Burrows–Wheeler Aligner (BWA-MEM; Sentieon), sequenced data were aligned with genome reference to GRCh37 (hg19) (15). Duplicate marking, indel realignment, baseline quality recalibration, and variant calling were performed using the Genome Analysis Toolkit (GATK) algorithms (Sentieon) (16). Variant annotation, filtering, and interpretation were performed with the VarSome Clinical platform (Spatter). Variants in the coding and splicing regions and known pathogenic variants in the noncoding regions were included in the analysis. Variants with minor allele frequencies (below 1%) in the gnomAD database were evaluated. Filtering and prioritization of potentially disease-causing variants were performed based on the inheritance pattern, information in the ClinVar database, Human Phenotype Ontology (HPO) terms associated with the patient's findings, and in silico pathogenicity estimates. Variants were classified according to the ACMG guidelines (17) and ClinGen recommendations (18), pathogenic (P), and likely pathogenic (LP), and clinically relevant variants of uncertain significance (VUS) associated with the patient's clinical findings were reported.

### WGS

Genomic DNA was fragmented enzymatically, and libraries were generated using polymerase chain reaction-mediated addition of Illumina-compatible adapters. Libraries were sequenced with paired ends on an Illumina platform to achieve an average depth of coverage of ∼30×. Alignment of the GRCh37/hg19 genome and human mitochondrial DNA (NC_012920) to the revised Cambridge Reference Sequence (rCRS), variant calling, annotation, and extensive variant filtering were applied through an inhouse bioinformatics pipeline. The copy number variant (CNV) recall was based on Illumina's DRAGEN pipeline. All variants in the GnomAD database with minor allele frequency of <1% and disease-causing variants reported in the Human Gene Mutation Database, ClinVar, and CentoMD were evaluated. During the evaluation, the entire gene region was examined for candidate variants associated with the phenotype in exons and in ±20 intronic regions. All potential patterns were considered for the inheritance pattern. Furthermore, family history and clinical information were used to assess the pathogenicity and probability of the disease-causing effect of the detected variants. Variants were divided into five classes (pathogenic, possibly pathogenic, of uncertain clinical significance, possibly benign, and benign) according to the ACMG guidelines. All potential variants associated with the patient's phenotype were reported. CNVs of uncertain clinical significance were not reported. Variants with heteroplasmy levels of up to 15% were reported as mitochondrial variants. Centogene has established specific quality criteria and validation processes for variants detected by NGS. Variants with poor sequence quality and/or indeterminate zygosity were confirmed by orthogonal methods. As a result, a specificity of >99.9% was guaranteed for all reported variants.

### Chromosomal microarray

Technical work was conducted using the Affymetrix Cytoscan 750 K Microarray Platform. Findings that fell within the resolution thresholds (at least 25 markers for decreases of 100 kb and greater and increases of 200 kb and greater in known genomic areas) were analyzed using filters in the “Chromosome Analysis Suite (CHAS)” analysis program. Findings outside the reporting standards; absence of heterozygosity regions; additional mosaic findings; findings below the resolution threshold overlapping with the Online Mendelian Inheritance in Man (OMIM) genes, above-threshold findings without the OMIM genes, the OMIM genes not detected but findings in loci associated with the phenotype; findings that cannot be directly associated with the reason for referral; and results other than the OMIM gene point mutations, small deletions or duplications, low rate mosaicism, and balanced chromosomal changes such as reciprocal and the Robertsonian translocations were not evaluated. Whether the findings were familial or not was determined only through the studies requested by the parents. When required, the segregation analysis of the CNVs detected in the mother and father via array analysis was investigated.

GRCh37/hg19 was used as the reference genome in the analysis. The ClinGen, Decipher, UNIQUE, OrphaNet, DGV, ClinVar, OMIM®, dbSNP, and HPO databases associated with the patient's clinical findings were used to assess the variants. Detected variants were assessed using the patient's clinical findings.

Detected variants were classified according to the ACMG-ClinGen Variant Classification Guidelines. Variants classified as pathogenic, possibly pathogenic, and of uncertain clinical significance associated with the patient's clinical findings were reported. Reported variants were named according to the ISCN 2020 nomenclature (19).

### Statistical analysis

The demographic and clinical characteristics of the patients evaluated in the study were analyzed via descriptive statistical analyses (number, percentage, mean, standard deviation, etc.). The comparisons of age, clinical findings, perinatal history, demographic characteristics, MRI, and EEG findings according to the genetic analysis results of the patients evaluated in the study were conducted using chi-square analysis. The significance level for all analyses was set at *p* < 0.05. The conformity of the data to normal distribution was checked with the values of kurtosis and skewness (±1.5). IBM SPSS 22.0 was used to conduct the analyses.

## Results

In total, 90 patients were included in the study. Of the 90 patients, 53 (58.9%) were male, and 37 (41.1%) were female. The age of the patients at the time they were seen at our clinic was 0.04–16 years. The mean age of the patients was 3.99 ± 2.71 years, and the median age was 2.71 years. Forty-seven patients were from Turkey; the remaining 43 patients were international (Table 1). EEG abnormalities were detected in a total of 58 (64.4%) patients. EEG was not available from four patients (P68, P77, P79, P81) (Table 2).

**Table 1:** Demographic characteristics of all patients.

Patient characteristics		N	%
Sex	Female	37	41.1
	Male	53	58.9
Country	Turkey	47	52.2
	Abroad	43	47.8
Age at admission (years)	<2	36	40
	2–5	29	32.2
	≥5	25	27.8
Age at seizure onset (years)	No seizures	28	31.1
	<2	48	53.3
	≥2	14	15.6

**Table 2:** Electroencephalography findings.

EEG findings	Epilepsy only		Epilepsy+DD		DD	
	*N*	%	*N*	%	*N*	%
FED	5	55.6	28	52.9	3	10.8
G	2	22.2	9	17	0	0.0
FED+G	1	11.1	5	9.4	0	0.0
SWA	0	0.0	2	3.8	1	3.5
BS	0	0.0	2	3.8	0	0.0
Normal	1	11.1	7	13.2	20	71.4

BS, burst suppression; DD, developmental delay; EEG, electroencephalography; FED, focal epileptic discharge; G, generalized epileptic discharge; SWA, slow wave activity.

Cranial MRI was abnormal in 44 patients (48.8%). The MRI results were abnormal in 27 (50%) of 54 patients with P/LP variants and clinically relevant VUS variants. MRI was not available in two patients (P68, P82) (Table 3).

**Table 3:** Magnetic resonance imaging findings of all patients.

MRI findings	N*	%
Corpus callosum atrophy	15	16.6
Ventriculomegaly	13	14.4
White matter changes	12	13.3
Cerebral atrophy	10	11.1
Cerebellar atrophy	9	10
Basal ganglia and/or thalamus lesions	6	6.6
Hypoxic ischemic brain injury	5	5.5
Hydrocephalus	3	3.3
Cortical dysplasia	2	2.2
Cerebral venous sinus thrombosis	1	1.1
Intracranial hemorrhage	1	1.1
Hypothalamic hamartoma	1	1.1
Holoprosencephaly	1	1.1
Optic nerve atrophy	1	1.1

MRI, magnetic resonance imaging.

*Some patients had more than one finding.

In 15 (16.6%) patients, parental consanguinity/origin from same village was reported. There was first-degree cousin marriage between the parents in seven patients (7.8%), and second-degree cousin marriage between the parents in five patients (5.6%). In total, 24 patients (26.7%) had a family history of similar diseases. Thirty-one patients (34.4%) had abnormal perinatal history, such as asphyxia, infection, and hospitalization in the neonatal intensive care unit (Supplementary Table S1).

**Figure 1:**
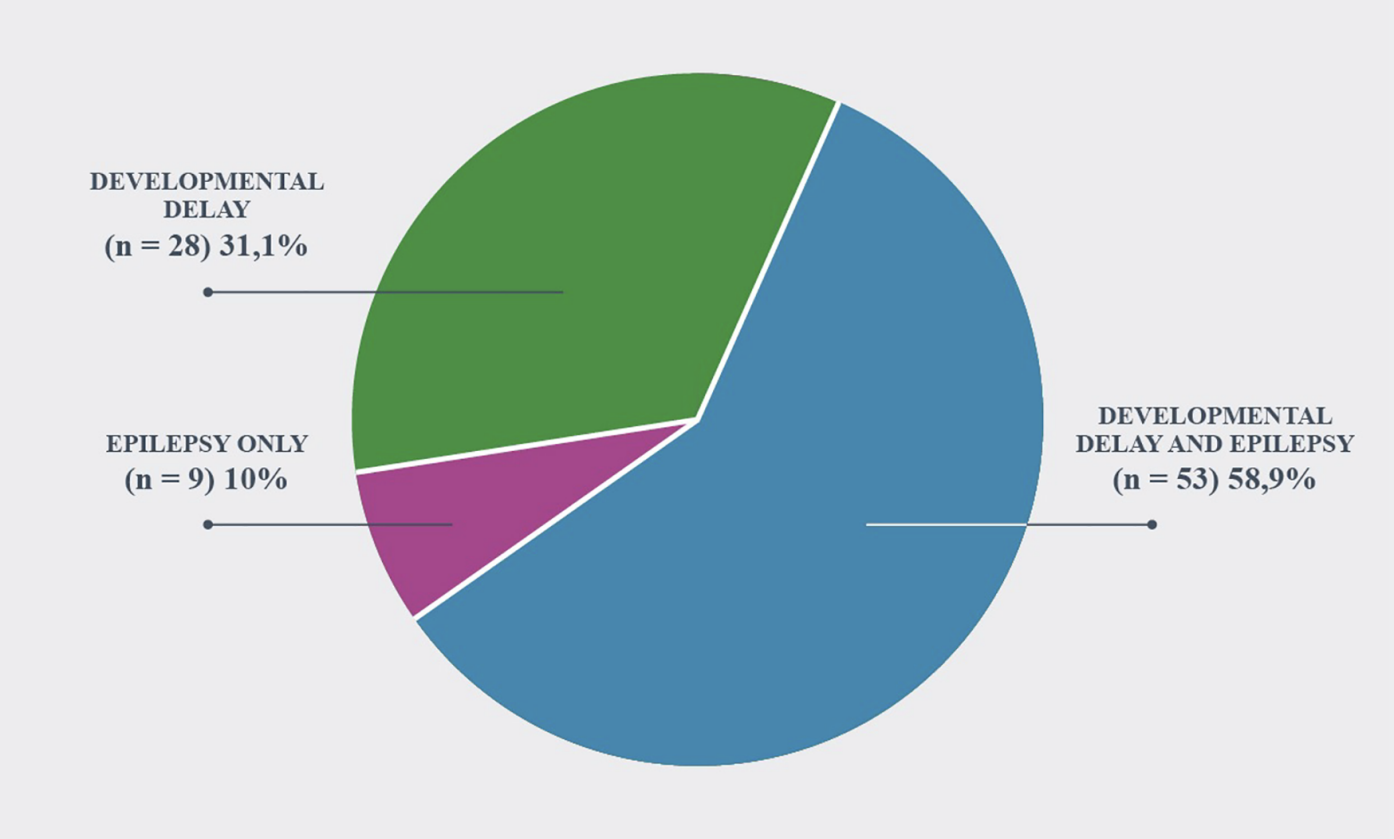
Phenotype of patients.

The patients were classified into three main groups; epilepsy only (*n* = 9; 10%), DD and epilepsy (*n* = 53; 58.9%), and only DD (*n* = 28; 31.1%) (Figure 1).

### Molecular findings

**Figure 2:**
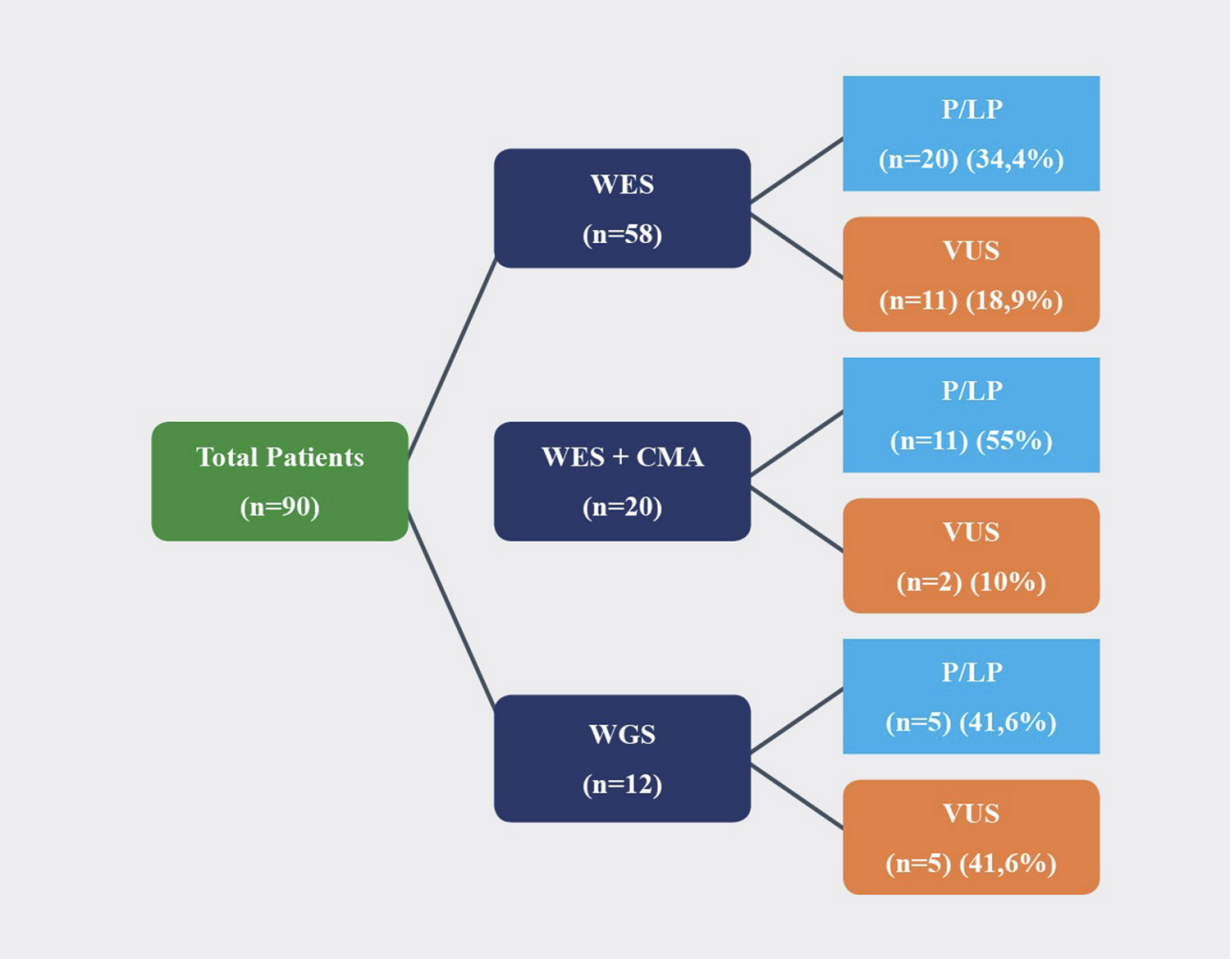
Molecular analysis results of patients.

Among all patients (*n* = 90), WES was studied in 58 patients, WGS was studied in 12 patients, and WES and CMA were studied in 20 patients. Trio-WES was performed in four, and trio WGS was performed in two families. Among the 58 patients who had undergone WES, P/LP variants were detected in 20 (34.4%), and clinically relevant VUS was detected in 11 (18.9%). Among the 12 patients who had undergone WGS, P/LP variants were detected in 5 (41.6%), and VUS was detected in 5 (41.6%). Among the 20 patients who had undergone WES and CMA, P/LP variants were detected in 11 (55%), and VUS was detected in 2 (10%) (Figure 2). A molecular diagnosis enabling a potential treatment option was established 12 patients (13.3%), (Table 4).

**Table 4:** Molecular diagnosis enabling potential treatment in epilepsy only and epilepsy+DD groups.

P	S	Genetic test	Gene	Zygosity	I	ACMG	D	T
P2	M	WES, CMA	*PRRT2* (NM_001256442.1.)	Heterozygous	AD	P	BFIS	OXC
P3	M	WES	*PRRT2* (NM_145239.3)	Heterozygous	AD	P	BFIS	PB
P4	M	Trio-WGS	*KCNQ2*	Heterozygous	AD, *de novo*	LP	DEE	PB, CBZ
P5	M	WES	*SCN2A* (NM_001040143.2)	Heterozygous	AD, *de novo*	LP	BFIS	LEV,CBZ
P10	M	WES	*FOLR1* (NM_016729.3)	Homozygous	AR	VUS	NCFTD	CZP, TPM, folinic acid
P11	M	Trio-WES	*S CN1A (NM_001353958.2)*	Heterozygous	AD, *de novo*	LP	Dravet Syndrome	VPA, TPM
P41	M	WES CMA	*SCN1A* (NM_001202435.1)	Heterozygous	AD	LP	Dravet Syndrome	LEV, TPM, PB, STP, FFA
P27	F	WES	*SLC2A1* (NM_6516.4)	Heterozygous	AD, *de novo*	VUS	GLUT-1 deficiency	KD, LEV, CLB
P25	M	WES CMA	*PNPO* (NM_018129.3)	Homozygous	AR	P	PNPOD	P5P, VPA
P35	M	WES	*ALDH7A1* (NM_001201377.2)	Homozygous	AR	P	EPD	TPM, PB, folic acid, L-carnitine, P5P
			*SLC22A5* (NM_003060.4)	Homozygous	AR	LP	CDSP	
P33	F	WES	*KCNQ2* (NM_172107.4)	Heterozygous	AD	P	DEE 7	VGB, CBZ

BFIS, benign familial infantile epilepsy; CBZ, carbamazepine; CDSP, primary carnitine deficiency; CLB, clobazam; CZP, clonazepam; D, diagnosis; DEE, developmental and epileptic encephalopathy; EPD, pyridoxine-dependent epilepsy; F, female; FFA, fenfluramine; I, inheritance; KD, ketogenic diet; LEV, levetiracetam; M, male; NCTFD, cerebral folate deficiency; OXC, oxcarbazepine; P, patient; PB, phenobarbital; PNPOD, pyridoxine 5ʹ-phosphate oxidasedeficiency; P5P, pyridoxal 5ʹ-phosphate; S, sex; STP, stiripentol; T, treatment; TPM, topiramate; VGB, vigabatrin; VPA, valproic acid.

In our cohort, several genes were frequently implicated in cases of genetic epilepsy and developmental delay, including *PRRT2* (n:2), *KCNQ2* (n:2), *SCN1A* (n:2), *PLA2G6* (n:2), and *KDM6A* (n:2). Among molecularly diagnosed cases *SCN1A, SCN2A, KCNQ2, SCN8A*, and *ATP1A2* were identified as channelopathy-related genes, representing 7 patients. While channelopathies were less frequent compared to other conditions in the cohort, their distinct clinical characteristics warrant further attention.

Developmental delay was present in 71.4% of patients with channelopathies, slightly lower than the 93.6% observed in patients with other conditions. This lower prevalence may reflect the primary impact of channelopathies on seizure activity and excitability rather than broader neurodevelopmental processes. Similarly, dysmorphic features were observed in 14.2% of patients with channelopathies, significantly less frequent compared to 36.1% in other genetic conditions, highlighting the subtler phenotypic manifestations of channelopathies.

Abnormal MRI findings were detected in 28.5% of patients with channelopathies, which was markedly lower than the 53.1% observed in patients with other genetic conditions. This suggests that structural brain abnormalities are less commonly associated with channelopathies, reinforcing their functional nature. In contrast, EEG abnormalities were present in 71.4% of patients with channelopathies, slightly higher than the 61.7% seen in other genetic conditions. This reflects the prominent role of electrical disturbances in the pathophysiology of channelopathies.

Table 5 provides a detailed summary of the molecular findings in the diagnosed cohort, further illustrating the differences in clinical and genetic characteristics between channelopathies and other neurodevelopmental conditions.

**Table 5:** Molecular findings in the diagnosed cohort.

P	G	Molecular test	Gene (Transcript)	Nucleotide	Protein	Zygosity	Inheritance	Variant	ACMG	Novel/Previously reported variant	Diagnosis (#OMIM number)
Epilepsy only											
P2	M	WES CMA	*PRRT2* (NM_001256442.1.)	c.629dup	p.Ala211Serfs*14	Het	AD	fs	P	Clinvar 31171	BFIS, 2 (#605751)
P3	M	WES	*PRRT2* (NM_145239.3)	c.694dup	p.Arg217Profs*8	Het	AD	fs	P	Clinvar 65758	BFIS, 2 (#605751)
P4	M	Trio-WGS	*KCNQ2, EEF1A2*	20q13.33 (62074225_62129854)x1		Het	AD, *de novo*	CNV	LP	Novel	DEE 7 (#613720), BFNS1 (#121200), DEE 33(#616409)
P5	M	WES	*SCN2A* (NM_001040143.2)	c.379G>C	p.Val127Leu	Het	AD, *de novo*	mis	LP	Novel	DEE, 11(#613721), BFIS, 3(#607745)
P6	F	WES	*NBEA* (NM_015678.5)	c.1370G>T	p.Cys457Phe	Het	AD	mis	VUS	Novel	NEDEGE (#619157)
Epilepsy and developmental delay											
P10	M	WES	*FOLR1* (NM_016729.3)	c.322G>A	p.Glu108Lys	Hom	AR	mis	VUS	Clinvar 450195	NCFTD (#613068)
P11	M	Trio-WES	*SCN1A* (NM_001353958.2)	c.5341T>A	p.Tyr1781Asn	Het	AD, *de novo*	mis	LP	C Depienne et al. (20)	Dravet syndrome (#607208)
P12	M	WES	*KNL1* (NM_170589.4)	c.6700C>T	p.His2234Tyr	Hom	AR	mis	VUS	Novel	MCPH 4 (#604321)
P13	F	WES	*ATP1A2* (NM_000702.4)	c.2429G>A	p.Gly810Asp	Het	AD	mis	VUS	Novel	DEE 98 (#619605)
P14	F	WGS	22q11 del.	22q11 (18877539-21681527)x1		Het	AD	CNV	P	Decipher 472470 Akgun-Dogan et al. (21)	22q11 deletion syndrome (#611867)
P15	M	WES	*ASH1l* (NM_018489.3)	c.7189C>T	p.Arg2397*	Het	AD	ns	LP	HGMD CM1513932	IDD, AD 52 (#617796)
P16	M	Trio-WES	*PCK1* (NM_002591.4)	c.524G>A	p.Arg175Gln	Hom	AR	mis	VUS	Clinvar 1368065	PCKDC (#261680)
P17	M	WES CMA	*ABCC8* (NM_000352.6)	c.4432G>A	p.Gly1478Arg	Het	AD, paternal	mis	LP	Clinvar 434045	HHF 1 (#256450)
P18	M	WES CMA	*KAT6A* (NM_006766.4)	c.5828T>C	p.Met1943Thr	Het	AD	mis	VUS	Novel	MRD 32 (#616268)
			*MT-ATP8* (NC_012920.1)	c.98A>G*	p.Tyr33Cys	Homoplasmic	mt	mis	VUS	Novel	MC5DM2 (#516070)
P19	F	WGS	2q37del.	2q37.3 (237531981_242782258)x1		Het	AD	CNV	P	Decipher 324169 Akgun-Dogan et al. (21)	2q37 microdeletion syndrome (#600430)
			3q29 dup.	3q29.32q29 (176456169_197851444)x3		Het	AD	CNV	P	S. Goobie et al. (22) 2008 Akgun-Dogan et al. (21)	3q29 microduplication syndrome (#611936)
P20	F	WES	*GNB1* (NM_002074.5)	c.233A>G	p.Lys78Arg	Het	AD	mis	P	Clinvar 224714	IDD, AD 42 (#616973)
P21	M	WES	*UBA5* (NM_024818.6)	c.863T>C	p.Met288Thr	Hom	AR	mis	VUS	Clinvar 1429996	DEE 44(#617132)
P22	F	WES	*PACS2* (NM_001100913.3)	c.625G>A	p.Glu209Lys	Het	AD	mis	P	Clinvar 495141	DEE 66 (#618067)
P23	M	WES	*PPT1* (NM_000310.4)	c.538dupC	p.Leu180Profs*9	Hom	AR	fs	P	Clinvar 56201	NCLs 1(#256730)
P24	M	WES	*ADAM22* (NM_021723.5)	c.1312C>A	p.Pro438Thr	Hom	AR	mis	VUS	Marieke M. van der Knoop et al. (23)	DEE 61 (#617933)
P25	M	WES CMA	*PNPO* (NM_018129.3)	c.674G>A	p.Arg225His	Hom	AR	mis	P	Clinvar 223153	PNPOD (#610090)
P27	F	WES	*SLC2A1* (NM_6516.4)	c.275+1del	p.?	Het	AD, *de novo*	S	VUS	Clinvar 1048756	GLUT-1 deficiency syndrome (#606777)
P28	M	WES	*PLA2G6* (NM_003560.4)	22q13.1 (38484755_38511688)x0		Hom	AR	CNV	P	Neil V Morgan et al. (24)	INAD 1 (#26600)
P29	F	WES CMA	*KDM6A* (NM_001291415.2)	c.4180C>T	p.Leu1394Phe	Het	XLD	mis	VUS	Novel	Kabuki syndrome (#300867)
P30	M	WGS	*SPTBN4* (NM_020971.2)	c.455T>G	p.Leu152Arg	C/H	AR	mis	VUS	Akgun-Dogan et al. (21)	NEDHND (#617519)
			*SPTBN4* (NM_020971.2)	c.7149del	p.Pro2384Argfs*60	C/H	AR	fs	VUS	Clinvar 1709124 Akgun-Doganet al. (21)	NEDHND (#617519)
P31	F	WES CMA	*WWOX* (NM_016373.4)	c.716T>G	p.Leu239Arg	Hom	AR	mis	LP	Clinvar 871669	DEE 28 (#616211)
P32	F	WGS	*MACF1* (NM_012090.5)	c.3512T>C	p.Leu1171Pro	Het	AD	mis	VUS	Novel	LIS 9 (#618325)
P33	F	WES	*KCNQ2* (NM_172107.4)	c.793G>A	p.Ala265Thr	Het	AD	mis	P	Milh et al. (25)	DEE 7 (#613720), BFNS1 (#121200)
P34	F	Trio-WES	*GFAP* (NM_002055.5)	c.1217G>T	p.Arg406Met	Het	AD, *de novo*	mis	LP	Novel	Alexander Disease(#203450)
P35	M	WES	*ALDH7A1* (NM_001201377.2)	c.988C>T	p.Arg330*	Hom	AR	ns	P	Clinvar 1457545	EPD (#266100)
			*SLC22A5* (NM_003060.4)	c.1088T>C	p.Leu363Pro	Hom	AR	mis	LP	Clinvar 25408	CDSP (#212140)
P36	F	WES	*PLA2G6* (NM_003560.4)	c.753dup	p.Asn252Glnfs*130	Hom	AR	fs	LP	Sallevelt et al. (26)	INAD 1 (#256600)
P37	F	WGS	*NAGA* (NM_000262.2)	c.973G>A	p.Glu325Lys	C/H	AR, paternal	mis	P	Clinvar 18162 Akgun-Dogan et al. (21)	NAGA deficiency type 3 (#609241)
			*NAGA* (NM_000262.2)	c.103C>T	p.Arg35Cys	C/H	AR, *de novo*	mis	VUS	Clinvar 429497 Akgun-Dogan et al. (21)	NAGA deficiency type 3 (#609241)
P39	M	Trio-WGS	*LSS* (NM_001001438.2)	c.1970T>G	p.Met657Arg	C/H	AR	mis	VUS	Novel	APMR 4 (#618840)
			*LSS* (NM_001001438.2)	c.2123G>T	p.Arg708Met	C/H	AR	mis	VUS	Novel	APMR 4 (#618840)
			*TUBB* (NM_001293212.1)	c.334T>G	p.Phe112Val	Het	AD,*de novo*	mis	VUS	Novel	CDCBM 6 (#615771)
P40	M	WES	*CUX1* (NM_001913.5)	c.1733_1734dupGC	p.Leu579Alafs*18	Het	AD	fs	LP	Novel	GDDI (#618330)
P41	M	WES CMA	*SCN1A* (NM_001202435.1)	c.1070 del	p. Asn357llefs*22	Het	AD	fs	LP	Novel	Dravet syndrome (#607208)
P44	F	WES	*SETD1A* (NM_014712.3)	c.2642G>T	p.Gly881Val	Het	AD,maternal	mis	VUS	Novel	EPEDD (#618832)
Developmental delay											
P63	M	WES	*BCL11A* (NM_022893.4)	c.1486G>T	p.Glu496Ter	Het	AD,*de novo*	ns	P	Novel	Dias-Logan Syndrome (#617101)
			*SCN8A* (NM_014191.4)	c.1743_1744delTG	p.Asn581LysfsTer5	Het	AD,*de novo*	fs	P	Novel	BFIS 5(#617080), CIAT (#614306), DEE 13(#614558)
P64	M	WGS	*THOC2* (NM_001081550.1)	c.521A>G	p.Lys174Arg	Hemizygous	XLR, maternal	mis	VUS	Novel	XLID 12 (#300957)
P65	M	WES CMA	3q13.31 mikrodel.	3q12.1q13.32 (98920626_118556822)x1		Het	AD	CNV	P	Molin et al. (27)	3q13.31 deletion syndrome (#615433)
P66	M	WES	*BCL11B* (NM_138576.4)	c.2438_2459del	p.Val813Alafs*24	Het	AD,*de novo*	fs	LP	Goos et al. (28)	IDDSFTA (#606558)
P67	F	WGS	*ERCC6* (NM_000124.3)	c.543+1G>T	p.?	Hom	AR	S	LP	Clinvar 974894 Akgun-Dogan et al. (21)	Cockayne syndrome (#133540)
P68	M	Trio-WES	*ECEL1* (NM_004826.4)	c.1507-9G>A	p.?	C/H	ARl	S	LP	Clinvar 210907	Arthrogryposis, distal type (#615065)
			*ECEL1* (NM_004826.4)	c.1630C>T	p.Arg544Cys	C/H	AR	mis	VUS	Clinvar 816801	Arthrogryposis, distal type (#615065)
P69	F	WES CMA	*KDM6A*	Xp11.3 (44733823_44837306)x1		Het	XLD	CNV	P	Clinvar 564836	Kabuki syndrome (#300867)
P70	M	WES CMA	*TCF 20* (NM_005650.4)	c.5452G>T	p.Glu1818*	Het	AD, *de novo*	ns	P	Novel	DDVIBA (#618430)
P71	F	WES	*DHCR7* (NM_001360.3)	c.452G>A	p.Trp151*	C/H	AR	ns	P	Clinvar 21273	Smith-Lemli-Opitz Sendromu (#270400)
			*DHCR7* (NM_001360.3)	c.1295A>G	p.Tyr432Cys	C/H	AR	mis	P	Clinvar 1076651	Smith-Lemli-Opitz Syndrome (#270400)
P72	F	WES	*GCDH* (NM_000159.4)	c.383G>A	p.Arg128Gln	Het	AR	mis	LP	Clinvar 189063	Glutaric acidemia type 1(#231670)
			*GCDH* (NM_000159.4)	c.1282A>G	p.lle428Val	Het	AR	mis	VUS	Novel	Glutaric acidemia type 1(#231670)
P73	M	WES	*DARS1* (NM_001349.4)	c.606C>G	p. lle202Met	Hom	AR	mis	VUS	Novel	HBSL (#615281)
			*ERLIN2* (NM_007171.2)	c.78A>G	p.Ile26Met	Het	AD	mis	VUS	Novel	SPG 18 (#611225)
P74	F	WES CMA	*ZEB2* (NM_014795.4)	c.1091C>G	p.Ser364*	Het	AD	ns	LP	Novel	Mowat-Wilson Syndrome (#235730)
P75	M	WES CMA	*SOX5*	12p12.1 (23805762_23967495)x1 del		Het	AD	CNV	P	Lamb et al. (29)	Lamb-Shaffer Syndrome (#616803)
P76	M	WES	*PANK2* (NM_153638.3)	c.676G>A	p.Val226Ile	Hom	AR	mis	LP	Akcakaya et al. (30)	NBIA 1 (#234200)
P77	F	WES	*PIK3R2* (NM_005027.4)	c.1117G>A	p.Gly373Arg	Mozaic	AD,*de novo*	mis	P	Clinvar 39808	MPPH 1 (#603387)
P78	M	WES	*SLC1A4* (NM_003038.5)	c.911G>A	p.Gly304Asp	Hom	AR	mis	VUS	Novel	SPATCCM (#616657)
P79	M	WGS	*LAMB1* (NM_002291.2)	c.4756A>T,	p.Thr1586Ser	C/H	AR	mis	VUS	Novel	LIS 5 (#615191)
			*LAMB1* (NM_002291.2)	c.4859T>C	p.Ile1620Thr	C/H	AR	mis	VUS	Clinvar 999947 Akgun-Dogan et al. (21)	LIS 5 (#615191)
P80	F	WES CMA	*KIF1A*	2q37.3 (240058543_242782258)x1 del		Het	AD	CNV	P	Decipher 411683, 277875	Nescav Syndrome (#614255)

Significant variants detected in the patients in our study are listed along with their characteristics. Variants reported in the literature are given with Decipher, HGMD, and ClinVar numbers. Variants that had not been reported previously in the literature are called new variants. These genetic variants’ classification detected according to by June 2024.

ACMG, American College of Medical Genetics and Genomics; APMR4, alopecia-intellectual disability syndrome 4; BFIS, benign familial infantile seizures; BFNS, benign familial neonatal seizures; CDCBM 6, cortical dysplasia, complex, with other brain malformations 6; CDSP, carnitine deficiency, systemic primary; CIAT, cognitive impairment with or without cerebellar ataxia; CNV, copy number variant; DDVIBA, developmental delay with variable intellectual impairment and behavioral abnormalities; C/H, compound heterozygous; DEE, developmental and epileptic encephalopathy; EPD, epilepsy, pyridoxine-dependent; EPEDD, epilepsy, early-onset, with or without developmental delay; F, female; fs, frameshift variant, G, gender; GDDI, global developmental delay with or without impaired intellectual development; GLUT1DS1, GLUT-1 deficiency syndrome; HBSL, hypomyelination with brainstem and spinal cord involvement and leg spasticity; Het, heterozygous; Hom, homozygous; HGMD, human gene mutation database; HHF, hyperinsulinemic hypoglycemia; IDD, AD, intellectual developmental disorder, autosomal dominant; IDDSELD: epilepsi ve konuşma geriliği ile birlikte zihinsel yetersizlik; IDDSFTA, intellectual developmental disorder with dysmorphic facies, speech delay, and T-cell abnormalities; INAD, infantile neuroaxonal dystrophy; LIS 9, lissencephaly 9 with complex brainstem malformation; M, male; MCPH 4, primary microcephaly 4; MC5DM2, mitochondrial complex 5 (ATP synthase) deficiency, mitochondrial type 2; mis, missense variant; MPPH1, megalencephaly-polymicrogyria-polydactyly-hydrocephalus syndrome 1; MRD 32, mental retardation, autosomal dominant; mt, mitochondrial; NBIA1, neurodegeneration with brain iron accumulation 1; NCFTD, neurodegeneration due to cerebral folate transport deficiency; NCLs, neuronal ceroid lipofuscinosis; NEDEGE, neurodevelopmental disorder with or without early-onset generalized epilepsy; NEDHND, neurodevelopmental disorder with hypotonia, neuropathy, and deafness; ns, nonsense variant; OMIM, online Mendelian inheritance in man; PCKDC, phosphoenolpyruvate carboxykinase deficiency, cytosolic; PNPOD, pyridoxamine 5′-phosphate oxidase deficiency; SPATCCM, spastic tetraplegia, thin corpus callosum, and progressive microcephaly; SPG 18, spastic paraplegia 18; S, splice-site variant; XLID 12, intellectual developmental disorder, X-linked 12.

Dysmorphic findings were significantly more frequent in the molecularly diagnosed group (P/LP/VUS variants), whereas other features, such as MRI abnormalities, neurobehavioral findings, and ocular findings, did not show a statistically significant difference (Table 6).

**Table 6:** Comparison of clinical characteristics according to patients' genetic results.

Clinical findings	Genetic results				*P*
	P/LP/VUS variants		–		
	*n*	%	*n*	%	
MRI findings	27	49.1	17	51.4	0.828
Spasticity	16	29.6	11	30.6	0.925
Hypotonia	20	37.0	7	19.4	0.074
Dystonia	4	7.4	1	2.8	0.348
Ataxia	0	0.0	2	5.6	0.080
ADHD	8	14.8	3	8.3	0.358
Autism	1	1.9	1	2.8	0.720
Microcephaly	8	14.8	5	13.9	0.903
Macrocephaly	4	7.4	0	0.0	0.095
Dysmorphism	18	33.3	2	5.6	0.002
Strabismus	4	7.4	2	5.6	0.730
Nystagmus	0	0.0	1	2.8	0.218
Optic atrophy	2	3.7	1	2.8	0.811
Coloboma	1	1.9	0	0.0	0.412
Cataract	1	1.9	1	2.8	0.770
Visual impairment	1	1.9	0	0.0	0.412
Microphthalmia	0	0.0	1	2.8	0.218
Swallowing difficulty	7	13.0	4	11.1	0.793
Hearing loss	2	3.7	1	2.8	0.811
Status epilepticus	2	3.7	1	2.8	0.811
Cleft palate lip	1	1.9	1	2.8	0.770
Hypothyroidism	3	5.6	2	5.6	0.999
Hydrocephalus	2	3.7	1	2.8	0.811
Sialorrhea	4	7.4	1	2.8	0.348

ADHD, Attention deficit and hyperactivity disorder; MRI, Magnetic resonance imaging.

## Discussion

Several epilepsy phenotypes exhibit chromosomal imbalances or alterations in genes encoding ion channels and transcription factors. Genetic testing is crucial for elucidating the underlying cause in patients with epilepsy and DD (31). Despite advanced diagnostic technologies nearly half of the affected individuals remain undiagnosed.

In the literature, the rate of diagnosis of DD and epilepsy by utilizing WES is reported to be between 25% and 55% (32–36). Hiraide et al., have reported that WES, including CNV analysis, resulted in a diagnostic rate of 53.5% (37). In our study, the percentage of LP/*P* variants with WES was 34.4%. Clinically relevant VUS were observed in an additional 18.9%, providing a similar diagnostic rate (53.3%) despite absence of CNV analysis. Similarly, the diagnostic rate of WGS in DD and epilepsy cohorts is reported to be 21%–50% (38–42). Among the 12 patients who had undergone WGS, 5 (41.6%) had P/LP variants, and 5 (41.6%) had clinically relevant VUS. WGS was performed in patients without prior testing. Two out 5 P/LP WGS results showed CNV as the underlying cause which would have been missed by WES alone. WGS shortened the diagnostic duration in these patients as first-tier genetic test.

The findings of our study indicate that the most frequently observed genes are consistent with those reported in the literature (11–14). Furthermore, although a direct comparison is not feasible due to the significant differences in the numbers, channelopathy-related genes stood out prominently in our study. Similarly, in the literature, channelopathy-related genes have also been highlighted as a significant group, in line with our data. Compared to other genetic etiologies underlying epileptic encephalopathies, channelopathies are less frequently associated with dysmorphic features and abnormal MRI findings, while EEG abnormalities are more prominently observed. This highlights the critical importance of genetic analysis in establishing an accurate diagnosis. Furthermore, genetic findings play a pivotal role in informing and tailoring treatment strategies (11, 13).

In this study, a molecular genetic diagnosis with available treatment was established in 13.3% of the patients. Only one patient with an actionable variant was left untreated due to absence of clinical seizures. Mahler et al. reported a treatment change rate of 6%. The higher rate of specific treatment changes in our study may be attributed to our patients having a more heterogeneous phenotype, as well as the emergence of new treatment options involving genes related to ion channels (31). By re-evaluating the previously negative WES data of patient P27, a *de novo* VUS variant in the *SLC2A1* gene was identified; this was followed by segregation analysis, which led to a diagnosis of GLUT-1 deficiency (43). Seizures were controlled using a ketogenic diet, demonstrating the importance of data reanalysis in treatment modifications.

Trio WES analysis was conducted in 4 families due to economic burden. Segregation analysis was performed following initial WES analysis. 20.7% of the diagnosed individuals showed de-novo monoallelic variants. Brunet et al. reported a much higher frequency of de- novo variants (40.3%) following trio WES in their study. The lower percentage of de-novo monoallelic variants in our cohort reflects the higher rate of consanguinity leading to a higher incidence of biallelic disorders (22.2%) in comparison to 16% reported by Brunet et al. (44).

Samati et al. reported abnormal MRI findings in one-third of individuals with epilepsy in a recent review. The most frequently observed abnormalities were encephalomalacia related to chronic infarcts, cerebral atrophy, disorders of neuronal migration, periventricular leukomalacia. Accompanying EEG finding was mainly focal discharges (78%) (45). In our study, corpus callosum atrophy (16.6%), ventriculomegaly (14.4%), and white matter changes (13.3%) were present in 48.8% of our patients and focal discharges were most common (40%) as well. Our cohort mainly consists of unexplained patients without evident focal lesions. The relatively lower percentage of focal discharges may be related to the lower number of focal lesions on MRI in our patient group who underwent genetic testing.

The main limitation of our study is the retrospective nature and heterogeneity of the genetic tests. Some patients had only WES, some had WES and CMA, and some had WGS. Therefore, the number of patients having each test was limited. However, our study adds novel genetic variants that have not been described previously in the literature. All patients received pre-test and post-test genetic counseling from two pediatric geneticists who also contributed to deep-phenotyping and reverse phenotyping of the diagnosed cohort. Patients with negative genetic results will be recalled for reanalysis yearly. Genetic counseling is provided by clinical geneticists in Turkey. Families with a diagnosis were counseled regarding recurrence risks and future reproductive options. This is the largest single center study with a multidisciplinary approach from our country.

In conclusión, NGS helps to determine the underlying molecular etiology of neurogenetic conditions. Moreover, an increasing percentage of patients receive personalized treatment after molecular diagnosis. WES and WGS increase the diagnosis rate and shorten the diagnostic odyssey for families when applied as first-tier genetic tests. Determining the genetic diagnosis of patients with epilepsy and/or DD using NGS is crucial in terms of an accurate prognosis, potential treatment methods, and a multidisciplinary approach involving molecular and clinical genetic expertise.

## Data Availability

The original contributions presented in the study are included in the article/Supplementary Material, further inquiries can be directed to the corresponding author.
