# Supplementary material for: Evaluation of the etiology of epilepsy and/or developmental delay in children via next-generation sequencing: a single-center experience
Source: Front Pediatr. 2025 Feb 27;13:1471965. doi: 10.3389/fped.2025.1471965 (PMC11904636; doi:10.3389/fped.2025.1471965)
Supplement: Supplementary file 1 [file Table1.docx]

| **Table S1.** Clinical, MRI, and EEG findings of patients with P/LP/VUS variants confirmed in genetic tests. | | | | | | | |
| --- | --- | --- | --- | --- | --- | --- | --- |
| **P** | **S** | **Age (year)** | **Country** | **Age at first seizure (year)** | **Symptom and Findings** | **EEG** | **MRI Findings** |
| **Epilepsy only** | | | |  |  |  |  |
| P2 | M | 0-2 | Turkey | 0.41 | Epilepsy, positive family history (mother) | FED, G | N |
| P3 | M | 0-2 | Turkey | 0.25 | Epilepsy, positive family history (father) | N | N |
| P4 | M | 0-2 | Romania | 0 | Epilepsy | FED | N |
| P5 | M | 0-2 | Turkey | 0.08 | Epilepsy, partial cleft palate lip | FED | N |
| P6 | F | ≥2-5 | Turkey | 3 | Epilepsy, positive family history | FED | N |
| **Epilepsy and Developmental Delay** | | | | |  |  |  |
| P10 | M | ≥5 | Algeria | 5 | Epilepsy, GDD (language, motor, cognitive), sialorrhea, gaze palsy, 2nd degree cousin marriage | G | WMC, CBA |
| P11 | M | ≥2-5 | Sudan | 0.33 | GDD (language, cognitive) Epilepsy,  ADHD, sialorrhea | N | N |
| P12 | M | 0-2 | Turkey | 0.75 | Epilepsy, microcephaly, GDD (language, motor, cognitive), dysmorphism, strabismus, pes equinovarus, spasticity, 2nd degree cousin marriage, perinatal history | FED, G | CCA, VMG |
| P13 | F | 0-2 | Turkey | 0 | Epilepsy, microcephaly, GDD (language motor, cognitive), dysphagia, hypotonia, perinatal history,  deceased sibling, 1st degree cousin marriage | FED | CCA, CBA |
| P14 | F | ≥2-5 | Ukraine | 0.91 | Epilepsy, GDD (language and cognitive), dysmorphism | SWA | N |
| P15 | M | ≥5 | Romania | 1.5 | Epilepsy, macrocephaly, GDD (language motor, cognitive) | G | N |
| P16 | M | ≥2-5 | Romania | 0.16 | Epilepsy, GDD (language and motor), toe syndactyly, dysmorphism, strabismus, | N | N |
| P17 | M | 0-2 | Bulgaria | 0 | Epilepsy, GDD (language and motor), microcephaly, hypotonia, spasticity, perinatal history | FED | CA, CCA |
| P18 | M | ≥5 | Albania | 0.58 | Epilepsy, GDD (language, motor, cognitive) perinatal history | G | CA, WMC, HIBI |
| P19 | F | 0-2 | Turkey | 0.66 | Epilepsy,  GDD (language and motor), Currarino syndrome, hypotonia, perinatal history, cardiac defect (large secundum atrial septal defect) | FED | N |
| P20 | F | 0-2 | Turkey | 0.58 | Epilepsy, GDD (language, motor, cognitive), hypotonia,strabismus | FED | N |
| P21 | M | 0-2 | Bosnia and Herzegovina | 0.58 | Epilepsy, GDD (language, motor, cognitive) microcephaly, spasticity, gaze palsy | FED | CBA,CA, VMG, WMC |
| P22 | F | ≥2-5 | Turkey | 0 | Epilepsy, GDD (language and cognitive),  coloboma in the right eye | FED, G | N |
| P23 | M | ≥2-5 | Turkey | 3 | GDD (language, motor, cognitive), dysmorphism, Epilepsy, hypotonia, autism, spasticity, microcephaly, dystonia, blindness, dysphagia, 2nd degree cousin marriage | FED | CA |
| P24 | M | 0-2 | Turkey | 0 | Epilepsy, status epilepticus, GDD (language motor, cognitive), gaze palsy, 1st degree cousin marriage | FED | CCA, BGTL |
| P25 | M | 0-2 | Kosovo | 0 | Epilepsy, hypotonia, GDD (language and motor),  hypothyroidism, dysmorphism, perinatal history | SWA | CCA,VMG,CA, OA |
| P27 | F | ≥5 | Turkey | 0.33 | Epilepsy, hemangioma under left eye, spasticity, GDD (language, motor, cognitive), dystonia | FED, G | N |
| P28 | M | ≥2-5 | Azerbaijan | 2.5 | Epilepsy, GDD (language, motor, cognitive), hypotonia, spasticity, 1st degree cousin marriage | FED | CBA |
| P29 | F | ≥5 | Serbia | 3 | Epilepsy, mental disability | FED | N |
| P30 | M | ≥2-5 | Turkey | 3 | Epilepsy, GDD (language and motor), 1st degree cousin marriage | FED | N |
| P31 | F | 0-2 | Kazakhistan | 0.33 | Epilepsy, GDD (language, motor, cognitive), dysmorphism, hypotonia, achalasia, optic atrophy, perinatal history, parents from the same region | FED | N |
| P32 | F | ≥5 | North Macedonia | 5 | Epilepsy, GDD (language, motor, cognitive), spasticity, tremor, epileptic encephalopathy, dysphagia, positive family history, epilepsy in sibling, sialorrhea | FED, G | CBA |
| P33 | F | 0-2 | Bulgaria | 0 | Epilepsy, GDD (language, motor, cognitive), hypotonia, dysphagia, hypothyroidism, strabismus | FED | VMG, CCA |
| P34 | F | 0-2 | Moldova | 0.5 | Epilepsy, hypotonia, GDD (language, motor, cognitive), hydrocephalus, dysphagia, GERD | N | WMC, VMG, HD |
| P35 | M | 0-2 | Turkey | 0 | Epilepsy, status epilepticus, dysmorphism, hypotonia, GDD (language, motor, cognitive), perinatal history, positive family history | MED | CCA,CVST |
| P36 | F | ≥2-5 | Turkey | 2 | Epilepsy, GDD (language, motor, cognitive) hypotonia, positive family history, similar disease in sibling, 1st degree cousin marriage | FED | CBA |
| P37 | F | ≥2-5 | Ukraine | 0.25 | Epilepsy, GDD (language, motor, cognitive), hypotonia, gaze palsy | FED | VMG, CCA |
| P39 | M | ≥2-5 | Kazakhistan | 0 | Epilepsy, GDD (language, motor, cognitive), hypotonia, spasticity, sparse hair, dysmorphism, perinatal history, similar disease in sibling, positive family history, | FED | CA, WMC, CCA |
| P40 | M | 0-2 | Bulgaria | 0.91 | Epilepsy, GDD (language, motor, cognitive), dysphagia, hypotonia, spastic quadriparetic, perinatal history | FED | VMG, WMC |
| P41 | M | ≥5 | Bosnia and Herzegovina | 0.58 | Epilepsy, mental disability, ADHD, | FED | N |
| P44 | F | ≥5 | Turkey | 1 | Epilepsy , absence epilepsy, ADHD, GDD (language and cognitive), hypothyroidism | G | N |
| **Developmental Delay** | | | | | |  |  |
| P63 | M | ≥5 | Georgia | - | GDD (language, motor, cognitive), ADHD, dysmorphism, prenatal history | N | N |
| P64 | M | ≥5 | North Macedonia | - | GDD (language and cognitive), EEG abnormality, ADHD, spasticity, obesity, cryptorchidism, similar disease in sibling, parents from the same region, positive family history | FED | N |
| P65 | M | ≥2-5 | Kosovo | - | GDD (language, motor, cognitive), febrile seizure, dysmorphism, spasticity, perinatal history | N | HIBI, CCA |
| P66 | M | 0-2 | Turkey | - | GDD (language, motor, cognitive), microcephaly, perinatal history, coronal synostosis | N | N |
| P67 | F | ≥5 | Romania | - | GDD (language, motor, cognitive), spasticity, deafness, congenital cataract, tremor, microcephaly, dysmorphism, perinatal history, parents from the same region | N | CA |
| P68 | M | ≥2-5 | Kosovo | - | Arthrogryposis, bilateral pes equinovarus, GDD (language and motor), toe syndactyly, dysmorphism | unavailable | unavailable |
| P69 | F | ≥5 | Turkey | - | Mental disability, dysmorphism, hyperextensible joints | N | N |
| P70 | M | ≥5 | Turkey | - | GDD (language. Motor, cognitive), EEG abnormality, macrocephaly, ADHD, deafness | FED | N |
| P71 | F | ≥2-5 | Turkey | - | GDD (language, motor, cognitive), toe syndactyly, dysmorphism, hypotonia | N | N |
| P72 | F | 0-2 | Turkey | - | GDD (language, motor, cognitive), hypotonia, dysphagia, spasticity, dystonia | N | BGTL |
| P73 | M | ≥5 | Afghanistan | - | GDD (motor), spasticity, dysmorphism, neurologic regression, 1st degree cousin marriage, positive family history | N | VMG, WMC, HIBI |
| P74 | F | ≥2-5 | Bosnia and Herzegovina | - | GDD (language, motor, cognitive), hypotonia, dysmorphism, | N | HH |
| P75 | M | ≥2-5 | Iraq | - | GDD (language and cognitive) ADHD, perinatal history | N | N |
| P76 | M | ≥5 | Turkey | - | GDD (language, motor, cognitive), ADHD, tortikolis, dystonia, spasticity, 2nd degree cousin marriage, similar disease in sibling, positive family history, perinatal history | N | BGTL |
| P77 | F | 0-2 | Turkey | - | GDD (language, motor, cognitive), macrocephaly, hydrocephalus, hyperextensible joint, dysmorphism, sialorrhea, positive family history | unavailable | CD, HD |
| P78 | M | 0-2 | Turkey | - | GDD (language, motor, cognitive), microcephaly, hypotonia, deceased sibling, similar disease in deceased sibling, 2nd degree cousin marriage | N | N |
| P79 | M | ≥2-5 | Djibouti | - | GDD (language, motor, cognitive), spasticity, hypotonia, deceased sibling | unavailable | BGTL |
| P80 | F | ≥5 | Ukraine | - | GDD (language, motor, cognitive), febrile seizure, polyneuropathy, macrocephaly, dysmorphism, hypotonia, club foot, DI, positive family history | N | VMG |
| Patients with significant variants in our study are listed together with their demographic and clinical characteristics, MRI, and EEG findings.  ADHD, attention deficit and hyperactivity disorder; BGTL, basal ganglia and/or thalamus lesions; BS, burst suppression; CA, cerebral atrophy; CBA, cerebellar atrophy; CCA, corpus callosum atrophy; CD, cortical dysplasia; CVST, cerebral venous sinus thrombosis; DI, diabetes insipidus; F, female; FEA, focal epileptic discharge; G, generalized epileptic activity; GDD, global developmental delay; GERD, gastroesophageal reflux disease; HD, hydrocephalus; HH, hypothalamic hamartoma; HIBI, hypoxic ischemic brain injury; HPE, holoprosencephaly; ICH, intracranial hemorrhage, M, male; MED, multifocal epileptic discharge; N, normal; OA, optic nerve atrophy; P, patient; S, sex; SWA, slow wave activity; VMG, ventriculomegaly; WMC, white matter changes. | | | | | | | |
